# Molecular Signaling Pathways and Therapeutic Targets in Hepatocellular Carcinoma

**DOI:** 10.3390/cancers12020491

**Published:** 2020-02-20

**Authors:** Manali Dimri, Ande Satyanarayana

**Affiliations:** Department of Biochemistry and Molecular Biology, Molecular Oncology & Biomarkers Program, Georgia Cancer Center, Augusta University, Room-CN3144, 1410 Laney Walker Blvd., Augusta, GA 30912, USA; sande@augusta.edu

**Keywords:** Hepatocellular carcinoma, signaling pathway, targeted therapy

## Abstract

Hepatocellular carcinoma (HCC) is a complex biological process and is often diagnosed at advanced stages with no effective treatment options. With advances in tumor biology and molecular genetic profiling, several different signaling pathways and molecular mechanisms have been identified as responsible for initiating and promoting HCC. Targeting these critical pathways, which include the receptor tyrosine kinase pathways, the Ras mitogen-activated protein kinase (Ras/Raf/MAPK), the phosphatidylinositol 3-kinase (PI3K)/AKT/mammalian target of rapamycin (mTOR), the Wnt/β-catenin signaling pathway, the ubiquitin/proteasome degradation and the hedgehog signaling pathway has led to the identification of novel therapeutics for HCC treatment. In this review, we elaborated on our current understanding of the signaling pathways involved in the development and initiation of HCC and anticipate the potential targets for therapeutic drug development.

## 1. Introduction

Hepatocellular carcinoma (HCC) accounts for 7% of all the cancers worldwide, and is the most common primary liver malignancy [1]. It is an aggressive tumor with a poor prognosis and is the second leading cause of cancer-related mortality. Over the past two decades, the incidence of HCC has doubled in the United States, Europe and many other countries. With 800,000 cases being diagnosed and an estimated 750,000 causalities annually worldwide [2]. The most common risk factors associated with the development of HCC include chronic hepatitis B and C virus infection. Approximately 75% of HCCs are associated with hepatitis infection, which might lead to a twenty-fold increase in HCC development [3]. Other major risk factors include chronic alcohol consumption, obesity, aflatoxin B1 (AFB1) exposure and nonalcoholic fatty liver disease (NAFLD) [4]. 

Current treatment options, including liver transplantation or surgical resections, can cure patients at early stages of HCC [5,6]. However, advanced HCC is diagnosed in 80% of patients, mainly due to a lack of symptoms and effective screening strategies for HCC [7]. Due to limited treatment options, systemic drug doses are often used, particularly when surgical sectioning is not possible. Unfortunately, HCC is also highly resistant to conventional chemotherapies with cytotoxic agents. Overall, the current treatment strategy for HCC can offer survival benefit, particularly to patients in early stages [8]. Only 30–40% of all the HCC patients are diagnosed as eligible for current treatment options due to late diagnosis [9,10].

HCC is a complex multistep biological process that involves genetic and epigenetic alterations [11]. There has been tremendous progress in our understanding of the critical oncogenic and tumor suppressor pathways involved in HCC [12,13,14,15,16]. The identification of several cell signaling pathways involved in tumor pathogenesis provide an opportunity to identify novel targets that can be utilized for therapeutic development. Targeted therapies act specifically on tumorigenesis regulating components, unlike broad-spectrum, conventional cytotoxic agents. Currently, the only approved drug for advanced HCC is Sorafenib, which partially targets the multi-kinases involved in advanced liver cancer [17,18]. The limitations of existing treatment options has led to a quest for the development of novel targeted therapies against HCC. In the last several years, there has been growing interest in molecular mechanisms playing a critical role in the development of HCC. Numerous signaling pathways have been observed to be dysregulated, either in response to viral infection or by exposure to toxic agents in HCC. Important gains in HCC biology suggest that alterations in multiple molecular signaling pathways such as Receptor tyrosine pathways, phosphatidylinositol 3-kinase (PI3K)/AKT/mammalian target of rapamycin (mTOR), Ras mitogen-activated protein kinase (Ras/Raf/MAPK), Wnt/β-catenin, Janus kinase (Jak)-signal transducer activator of transcription factor (Stat) (JAK/STAT), Hedgehog (HH) and Hippo signaling pathway are essential for carcinogenesis. With advances in our understanding of the molecular pathways involved in tumorigenesis, there is a growing interest for the development of novel therapeutics targets against the critical genes and signaling molecules regulating multiple pathways. In this review we have discussed recent advances in molecular mechanisms and the key genes underlying the development of HCC and in the advancement of novel strategies for HCC treatment.

## 2. Molecular Mechanisms and Targeted Therapies

### 2.1. The Receptor Tyrosine Kinase Pathways

**Figure 1:**
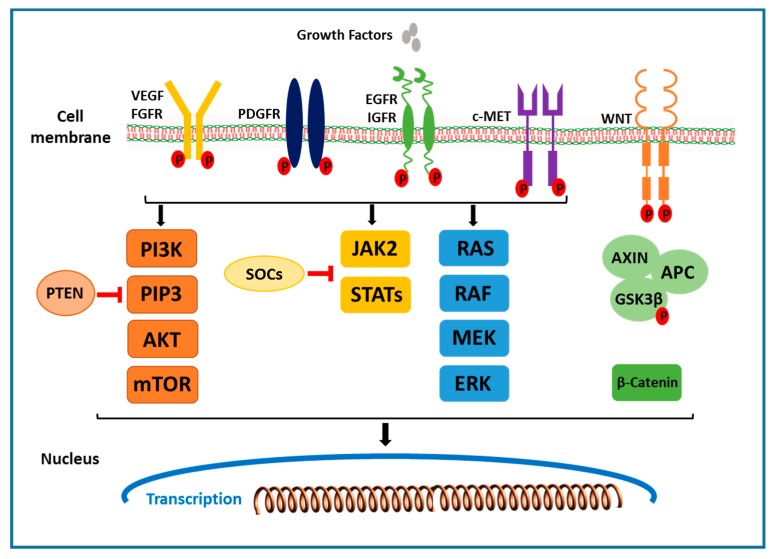
Schematic representation of major signaling pathways involved in hepatocellular carcinoma.

Receptors that activate multiple downstream signals in the receptor tyrosine kinase pathway include the epidermal growth factor (EGF) receptor, the fibroblast growth factor (FGF) receptor, the hepatocyte growth factor (HGF/ c-MET), the stem cell growth factor receptor c-KIT, the platelet-derived growth factor (PDGF) receptor, and the vascular endothelial growth factor (VEGF) receptor. Ligand binding and phosphorylation of these receptors, including the activation of the Grb2/Shc/SOS adapter molecule complex, lead to downstream activation of MAPK and PI3K pathways. Ras/Raf/MEK/ extracellular signal regulated kinase (ERK) pathway activation results in the activation of AP-1 transcriptional activators, cJun and cFos, which induce the transcription of genes that drive cell proliferation [19,20,21]. In HCC, initiation and progression of tumors is significantly affected by many growth factors, including the VEGF, HGF/c-MET, EGFR and IGF receptors (Figure 1).

#### 2.1.1. VEGF Receptor

The liver, being a highly vascular organ, depends on effective angiogenesis for cellular regeneration. Likewise, in HCC, efficient angiogenesis is critical for tumor growth vascular invasion and metastasis [22]. VEGF is a critical angiogenic factor and plays an important regulatory role in HCC. Three main subtypes of VEGF receptor are VEGF 1, 2 and 3. Among the three VEGF subtypes, VEGFR-2 appears to mediate most of the known cellular responses to VEGFs. In spite of the rich vascular supply, liver tumor displays abnormal vasculature such as arteriogenesis and capillarization. There is also abnormal blood flow due to immature liver tumor vessels, which are excessively leaky. This results in severe hypoxia in HCC, which in turn results in the induction of growth factors such as hypoxia-inducible factors 1 and 2 (HIF 1 and 2) and insulin like growth factor 2 (IGF2) [23]. Both HIF1 and IGF2 stimulate expression of VEGF and other growth factors, promoting tumor angiogenesis resulting in tumor progression and metastases. Studies in HCC cell lines and tissues as well as in the serum of patients with HCC have shown increased expression of VEGF and VEGF receptors (including VEGF-1, -2, -3) [24,25,26,27]. Increased expression of VEGF mRNA was found in liver tumors in the majority of the HCC patients and is correlated with tumor progression and poor survival [28]. The Hepatitis B x antigen was also associated with the up-regulation of VEGFR-3 [29]. Furthermore, disease recurrence, poor prognosis after resection, vascular invasion, and portal vein embolism are often correlated to high expression of VEGF [28]. Therefore, this suggests a clear regulatory role of VEGF mediated angiogenesis in HCC progression.

#### 2.1.2. EGFR and HGF/c-MET Signals

Mutations in the EGFR signaling pathway appear to be rare in HCC pathogenesis, but it plays a significant role in tumor angiogenesis and proliferation. The IGF signaling pathway is activated by the binding of ligands (IGF-1 and IGF-2) to the membrane bound receptor (IGF-1R) [30]. The IGF signaling pathway regulates several cellular processes, including proliferation, motility and apoptosis inhibition. Binding of the ligand to IGF-1R initiates receptor auto phosphorylation, followed by phosphorylation of intracellular targets (which includes insulin receptor substrates 1–4), which in turn results in the activation of downstream cellular effector factors and eventually activates the PI3K, protein kinase B and RAF/MEK/ERK pathway. The insulin like growth factor (IGF) signaling pathway is frequently dysregulated in HCC. The imbalance of IGF signaling occurs predominantly at the levels of IGF-2 availability. IGF-2 is overexpressed in human HCCs and this excess ligand availability leads to elevated receptor binding and further action on the MAPK and PI3K/AKT/mTOR pathways [31]. Furthermore, overexpression of *IGF-2* is correlated with increased cell proliferation in the HCC transgenic mice model [32], whereas *IGF-2* ablation reduced cell proliferation and increased apoptosis in SK-hep-1, Hep G2, and Hep 3B HCC cells [33].

HGF is a multifunctional cytokine that has been implicated in the invasion of tumor malignancies [34]. The HGF ligand exerts its effects by binding to the tyrosine kinase receptor c-MET. In addition to tissue regeneration, major functions of c-MET include cell proliferation, migration, survival, branch morphogenesis, and angiogenesis. HGF-induced activation of c-MET leads to self-phosphorylation of the c-MET receptor and further phosphorylation of adaptor proteins (including GRB2) and GAB1 (GRB2-associated -binding protein 1), which then activate downstream effector molecules (including phospholipase C, PI3K, and ERK). Moreover, c-MET dysregulation has been associated with multiple molecular genetic factors and its overexpression is correlated with a reduced five-year survival in HCC patients [35].

Given the contribution of this pathway during liver carcinogenesis, different compounds have been investigated at multiple levels to inhibit receptor tyrosine kinases. This includes inhibitors of the downstream activators like Raf and protein kinase C, as well as antibodies that bind to the receptors blocking ligand-receptor interactions. One such compound is BAY-439006, which inhibit multiple pathway components, Raf kinase and VEGF receptor, and initial clinical studies with this compound have shown partial responses in HCC patients [36]. Other inhibitor compounds relevant to HCC, for which preclinical studies have been published, include gefitini [37] EGFR tyrosine kinase inhibitor and FTY720, the PI3K inhibitor, which by downregulating the Rac GTP levels, suppresses the motility of the metastatic H2M HCC cell line [38]. Sunitinib is an oral and multi targeted tyrosine kinase inhibitor targeting VEGFR-1 and -2, PDGF receptor (PDGFR)-a and -b, the stem cell factor receptor c-KIT and others. Preclinical studies in multiple tumor cell lines have shown that the anti-angiogenic effects of sunitinib are mediated through VEGFR and PDGFR-β, however the primary target of sunitinib is likely to be VEGFR-2 [39]. Additional agents involved in inhibiting these signaling pathway components are described in Table 1.

### 2.2. RAF/MEK/ERK Signaling Pathway

The Raf/MEK/ERK is a ubiquitous signal transduction pathway that regulates crucial cellular processes, including cell proliferation, differentiation, angiogenesis and survival [60]. This pathway is activated by the binding of several growth factors to the receptors, triggering a series of specific phosphorylation events (Figure 1) [61]. This, in turn, activates an adapter molecule complex known as GRB2/SHC/SOS, which triggers the RAF/MEK/ERK pathway. The key molecular signal regulators within the Raf/MEK/ERK pathway is the small GTPase RAS and the serine/threonine kinase RAF. Intermediate signaling is regulated by mitogen/extracellular protein kinases MEK1 and MEK 2, which ultimately led to the phosphorylation of ERK1 and ERK2, the downstream signaling molecules. ERK1/2 regulate cellular activity by acting on a variety of substrates in the cytoplasm and nucleus [62]. The constitutive activation of the Ras/Raf/MAPK pathway was observed in HCC, suggesting its role in tumorigenesis. Furthermore, MEK1/2 phosphorylation was reported to be increased seven-fold in HCC tissues compared to the adjacent benign tissues [63]. In another study, increased expression and activity of signaling intermediates, such as phosphorylated ERK, has been observed in in vivo mouse HCC models and human HCC tissues [64,65]. Additionally, in solid tumors, RAF/MEK/ERK pathway is usually activated by two main mechanisms: (1) oncogenic mutations within the RAS gene, which leads to constitutive CRAF activation [60], and (2) constitutive CRAF activation resulting from a disorder in the overexpression of growth factors and their receptors [60]. Finally, it has been reported that the RAF/MEK/ERK pathway can also be activated by HBV infection in HCC. HBV expresses transcriptional activators by integrating into the host DNA, which eventually activates the RAF/MEK/ERK pathway [66]. Preclinical studies in HCC to target this pathway include oral inhibitors of MEK, a key enzyme in the Ras-Raf-MEK-extracellular signal regulated kinase (ERK) kinase pathway, which is constitutively active in variety of solid tumors. One of such compound is CI-1040, which has shown promising results in phase I, however, in phase II, it has demonstrated insufficient antitumor activity in patients with advanced breast cancer, colon cancer, non-small cell lung cancer and pancreatic cancer. Another compound PD 0325901, a second generation MEK inhibitor, entered clinical development, which has shown enhanced pharmacologic and pharmaceutical properties compared to CI-1040 [19] (Table 1). Erlotinib is a potent and reversible inhibitor of EGFR tyrosine kinase. In an in vitro study, it was shown to inhibit the RAF/MEK/ERK signaling pathway and blocked the signal transducer and activator of transcription-mediated signaling in Huh-7 and HepG2 cells [67]. The phase II study of 38 patients with unresectable or metastatic HCC, treated with Erlotinib, has shown preliminary anti-tumor activity [43]. Overall, the studies suggested that the RAF/MEK/ERK pathway works downstream of various growth factors and hence could be a promising therapeutic target in HCC.

### 2.3. PI3K/AKT/mTOR Signaling Pathway

In the PI3K/AKT/mTOR signaling pathway, the family of enzymes are activated by binding the of multiple growth factors (IGF and EGF) and cytokines to the receptor [68]. Upon activation, PI3K produces a lipid second messenger phosphoinositol triphosphate (PIP3) and related second messengers, which, in turn, activates AKT/protein kinase B (PKB). Akt is a serine threonine kinase downstream of PI3K. Activated Akt phosphorylates several cytoplasmic proteins and regulates a variety of critical cellular activities. One of the most important downstream effectors of Akt is a mammalian target of rapamycin (mTOR) subfamily of proteins (Figure 1) [69]. Akt activates the small G protein, Ras homolog enriched in brain (Rheb) by phosphorylating the tuberous sclerosis complex (TSC1/TSC2). Rheb, in its GTP-bound state, can activate mTOR. mTOR form a ternary complex to activate its signaling cascade. mTORC1 (mTOR Complex-1) contains mTOR, RAPTOR (Regulatory Associated Protein of mTOR), mammalian LST8/G-protein β-subunit like protein (mLST8/GβL), PRAS40 and DEPTOR. mTORC2 (mTOR Complex-2) consists of mTOR, rapamycin-insensitive companion of mTOR (Rictor), GβL, and mammalian stress activated protein kinase interacting protein 1 (mSIN1). Activated mTOR in turn regulates phosphorylation of the p70-S6 kinase, a serine threonine kinase and translational repressor protein PHAS-1/4E-BP which regulate cell cycle progression and proliferation. Another critical component of this pathway is Phosphatase and tensin homolog (PTEN). PTEN is a tumor suppressor gene that dephosphorylates PIP3 and blocks Akt activation and serves as a negative regulator of PI3K/AKT/mTOR signaling pathway (Figure 1) [19]. The PI3K/AKT/mTOR signaling pathway is the most critical and hyper activated in about ~50% of HCC. It controls several important cellular processes including cell growth, survival regulation and metabolism in multiple solid tumors [39,56]. It has been reported that anomalies in the PTEN function may lead to hyperactivation of the PI3K/AKT/mTOR pathway in HCC [70]. Importantly, mutation of *PTEN* gene or reduced PTEN expression is observed in approximately half of all HCC tumors, suggesting that inactivation of PTEN is involved in the pathogenesis of HCC [70,71]. Furthermore, in a transgenic hepatocyte-specific PTEN deficient mouse model, by 80 weeks of age, 66% of PTEN-deficient mice developed HCC [72]. In a microarray study, increased AKt phosphorylation on Ser473 was observed in 23% of HCC patients and was correlated with early HCC recurrence and poor prognosis [73]. mTORC1 and mTORC2 are the two critical components in this signaling pathway. mTORC1 is a downstream signal of AKT, which induce cell cycle progression by activating S6 kinase and regulate protein synthesis. Increased phospho-mTOR expression was detected in 15% of HCCs [74]. Furthermore, increased p70 S6 kinase expression is found in 45% of HCC patients, which is correlated with the mTOR phosphorylation [75]. In a recently reported study, reduced mTOR activity resulted in reduced cell proliferation in HepG2 and Hep3B HCC cell lines and reduced tumor growth in xenograft mouse models [74]. Recently, E2Fs has been shown to play a key role in the development of HCC. E2F family of transcription factors works downstream of cell cycle signaling and plays a crucial role in regulating cellular processes, such as cell proliferation, differentiation, senescense and apoptosis [76]. Studies in human HCC cell lines and transgenic mouse models have demonstrated that E2F1 inhibits c-Myc driven apoptosis via PIK3CA/Akt/mTOR and COX-2 pathways and promotes HCC development [77]. In another study, increased mRNA expression levels of E2Fs were reported in HCC patients and was correlated with poor prognosis and overall survival [78]. Currently, multiple agents targeting PI3K/AKT/mTOR pathways are under investigation for efficacy in treating HCC. Everolimus, mTOR inhibitor, treatment in Hep3B and SNU398 HCC cells has shown significantly reduced proliferative activity [79]. In precilincal HCC models, Everolimus has shown reduced tumor progression and improved survival [80]. In the phase III study, Everolimus was tested in patients with advanced HCC to assess it efficacy. However, no significant difference was observed between the treated and placebo treatment groups [81]. Rapamycin, a macrolide antibiotic with antifungal and immunosuppressive properties, inhibits the mTOR family kinases by binding to cytoplasmic FK506- binding protein-12 (FKBP12) and in complex with FKBP12. Rapamycin treatment in HCC cell lines reduced p70 S6K phosphorylation and significantly inhibited the HCC proliferation, suggesting a strong association between aberrant mTOR activity and HCC progression [75,82]. Taken together, these studies suggest a strong association between the PI3K/AKT/mTOR signaling pathway and HCC, and the inhibition of this pathway could be a strong HCC treatment strategy. 

### 2.4. Wnt/β-Catenin Signaling Pathway

In the Wnt/β-catenin signaling pathway, abnormal regulation of the transcription factor, β-catenin (a key component of WNT signaling pathway), play a major role in the development of early events in HCC. The Wnt/β-catenin signaling pathway is composed of Wnts, which are secreted cysteine-rich glycoprotein ligands [83,84]. The binding of these glycoprotein ligands to the members of frizzled family of cell surface receptors activates the receptor-mediated signaling pathway. Out of the nineteen Wnt ligands, binding to ten transmembrane frizzled receptors leads to the activation of either the canonical (β-catenin-dependent) or non-canonical (β-catenin-independent) Wnt pathway. In the absence of Wnt ligands, most β-catenin binds to E-cadherin in adherence junctions at the plasma membrane in most of the cells. Cytosol β-catenin forms complexes with adenomatous polyposis coli (APC) and AXIN1 or AXIN2, mediating sequential phosphorylation and degradation of β-catenin by casein kinase 1 and glycogen synthase kinase 3 β (GSK3β) [85]. In the presence of Wnt ligand, Wnt ligand binds to the cell surface Frizzled family receptor, resulting in phosphorylation and the inhibition of GSK3β, ensuring an increase in cytosolic β-catenin concentration (Figure 1). This leads to the translocation of β-catenin to the nucleus where it interacts with the TCF and LEF transcription factors and activates the transcription of genes involved in cell proliferation (*MYC*, *MYB*, *CJUN* and *CYD1*) angiogenesis (VEGF, c-MET), anti-apoptosis and the formation of the extracellular matrix [68,83,84]. In approximately 30–40% of human HCC patients, mutation in the β-catenin gene in 12% to 26% of human HCCs and mutations in AXIN 1 or AXIN 2 in 8% to 13% of human HCCs have been reported [86]. Importantly, increased levels of β-catenin in the cytoplasm and nucleus have been reported in approximately 50–70% of tumors in HCC [87], promoting proliferation and suppressing differentiation of tumor cells. Furthermore, studies in β-catenin transgenic mouse models, targeted disruption of the *Iqgap2* gene in mice, which is associated with Iqgap1 overexpression and the activation of β-catenin and cyclin D1, lead to the development of HCC. Breeding of *Iqgap2*-null mice with *Iqgap1*-null mice resulted in reduced HCC phenotype suggesting that *Iqgap2* acts as a tumor suppressor and its loss can lead to β-catenin activation and HCC development [88]. The critical role of Wnt in the regulation of liver regeneration and self-renewal of pluripotent stem cells and progenitor cells hence may be an ideal target for cancer cell therapy. Agents targeting Wnt pathways include small molecule inhibitors that block β-catenin TCF interaction, such as the fungal derivatives PKF115–854 and CGP04909035 or interaction of β-catenin with cAMP response element binding protein (CREB) binding protein (CBP), such as the small molecule ICG-001 [89], and therapeutic monoclonal antibodies against Wnts [90]. Currently several established drugs in clinical use have shown to inhibit Wnt signaling pathway, which includes nonselective cyclooxygenase (COX) inhibitors [19]. In a study, nuclear beta-catenin localisation and beta-catenin/TCF-regulated transcription of target genes was inhibited by nonsteroidal anti-inflammatory drug (NSAID) sulindac treatment, in vitro and in vivo [91]. In another study, treatment of SW948 and SW480 colorectal cancer cells with NSAIDs aspirin and indomethacin has shown reduced signaling activity of β-catenin [92]. The Wnt signaling pathway has a key role to play in HCC and the development of effective therapies would be beneficial to treat HCC.

### 2.5. JAK/STAT Pathway

The JAK/STAT pathway is activated by multiple cytokines, hormones and growth factors (Figure 1). It is involved in multiple functions such as differentiation, proliferation and apoptosis. In this pathway, the cytokines induce phosphorylation of the JAK family, which consists of four members Jak1, 2, 3 and Tyk2, followed by the activation of six members of the Stat family 1, 2, 3, 4, 5, and 6 [93,94]. Phosphorylation of tyrosine by JAKs results in the activation of this pathway. Activation of the JAK/STAT pathway is tightly controlled by three families of inhibitory proteins, the protein inhibitors of activated Stats (PIAS), the SH2- containing phosphatases (SHP), and the suppressors of cytokine signaling (SOCS) [95,96]. PIAS inhibits STAT-DNA binding and SH-2 is involved in suppression of a variety of cytokine signals. SOCS genes is mediated by STAT activation further inhibiting this pathway by binding to the phosphorylated JAKs and their receptors [97]. This prevents over activation of cytokine stimulated cells. Hence, SOCS functions as a part of negative feedback loop in this pathway. In HCC, a gain in the function mutations in JAKs has been detected to cause JAK/STAT pathway activation [98]. In 9% of patients with Hepatitis B-associated HCC, missense mutations in JAK1 have been identified. In vitro, these mutations increase phosphorylation of JAK1 and STAT3 and enable cytokine-independent growth [99]. STAT activation by JAK stimulation results in the initiation of cell proliferation, migration, differentiation and apoptosis. Importantly, inactivated JAK-binding proteins and activated JAK/STAT pathway have been reported in HCC [100,101]. A significant reduction in liver tumorigenesis, chemically induced by DEN and hepatocyte proliferation, was reported in JNK1 knockout mice models. It was reported that impaired proliferation was caused by an increased expression of p21, a cell cycle inhibitor. Furthermore, JNK activity ablation using inhibitor D-JNK1 reduced the growth of both xenografted human HCC cells and chemically induced mouse liver cancers [102]. These findings suggest a critical role played by JNK in HCC development and targeting JNK could be an ideal therapeutic approach to treat HCC. 2-(1-(4-(2-cyanophenyl)1-benzyl-1H-indol-3-yl)-5-(4-methoxy-phenyl)-1-oxa-3-azaspiro(5,5) undecane (CIMO) has been reported as a potent inhibitor of this pathway in HCC [57]. CIMO down-regulates constitutively active and inducible upstream kinases (STAT3) and the expression of target genes in vitro and in vivo and enhances cytotoxicity and deletes the nuclear pool of STAT3 in HCC (Table 1).

### 2.6. Hippo Signaling Pathway

Hippo signaling is an evolutionarily conserved pathway that modulates organ size and development by regulating cell proliferation, survival, differentiation and apoptosis [103]. In mammals, at the core of the Hippo pathway are serine/threonine kinases, sterile 20-related 1 and 2 kinases (MST1 and MST2), and large tumor suppressor 1 and 2 kinases (LATS1 and LATS2). MST1/MST2 forms a complex along with Salvador 1 (SAV1) to phosphorylate and activate LATS1/LATS2 [104]. Activated LATS1/LATS2 kinases, in turn, phosphorylate and inhibit the transcription co-activators Yes-associated protein (YAP) and transcriptional coactivator with PDZ-binding motif (TAZ). Tumor suppressor neurofibromin 2 (NF2) participates upstream of MST1/MST1 and LATS1/LATS2 kinases to inhibit YAP and TAZ activity by promoting the activation of the pathway. YAP/TAZ are transcriptional coactivators and does not comprise DNA binding domain. When LATS1/ LATS2 kinases are inactive, dephosphorylated YAP/TAZ translocate into the nucleus. In nucleus, YAP/TAZ interact significantly with TEA DNA-binding proteins (TEAD1–4) transcription factors and forms the YAP/TAZ-TEAD complex that mediate proliferative and prosurvival genes such as the connective tissue growth factor (CTGF), cysteine-rich angiogenic inducer 61 (CRY61) and others to promote cell survival, proliferation and growth [105]. In HCC, dysregulation of the Hippo pathway plays a crucial role in tumor development. Approximately 50% of human HCC patients show aberrant overexpression and nuclear localization of YAP [106]. In a study, YAP expression was evaluated in human HCC by immunochemistry, wherein out of 155 HCC samples tested, 63 samples (54%) showed strong YAP staining, whereas 95% of normal liver tissue samples showed weak staining, suggesting differences in YAP protein levels between normal and cancerous tissues. Additionally, analysis of HCC tumorous tissue showed significantly upregulated YAP protein and mRNA levels, as compared to the adjacent non-tumor tissue by 62% to 9%, respectively [107,108]. Notably, studies with YAP transgenic mouse showed YAP overexpression leads to HCC development, suggesting a direct connection between dysregulation of the Hippo pathway and liver tumorigensis [109]. Overall, these studies suggest that YAP activation plays a key role in human HCC and dysregulated hippo pathway could be common mechanism for YAP activation. Given its broad potential therapeutic relevance, there is a significantly growing interest in targeting the Hippo pathway. Verteporfin, also known as Visudyne, an FDA approved drug, has been widely reported as a Yap inhibitor [110]. It has been reported to reduce the expression of mRNAs of known Hippo pathway target genes such as CYR61, CTGF, AXL, and BIRC-5. Another YAP inhibitor, CA3, has been reported to modulate the YAP/TEAD transcriptional activity and decrease YAP expression [111]. Studies with Flufenamic acid has shown reduced cell growth, TEAD reporter activity and various Hippo pathway responsive genes [112]. These studies suggest that disruption of downstream transcription complexes could be a promising therapeutic approach.

### 2.7. Ubiquitin-Proteasome Pathway

**Figure 2:**
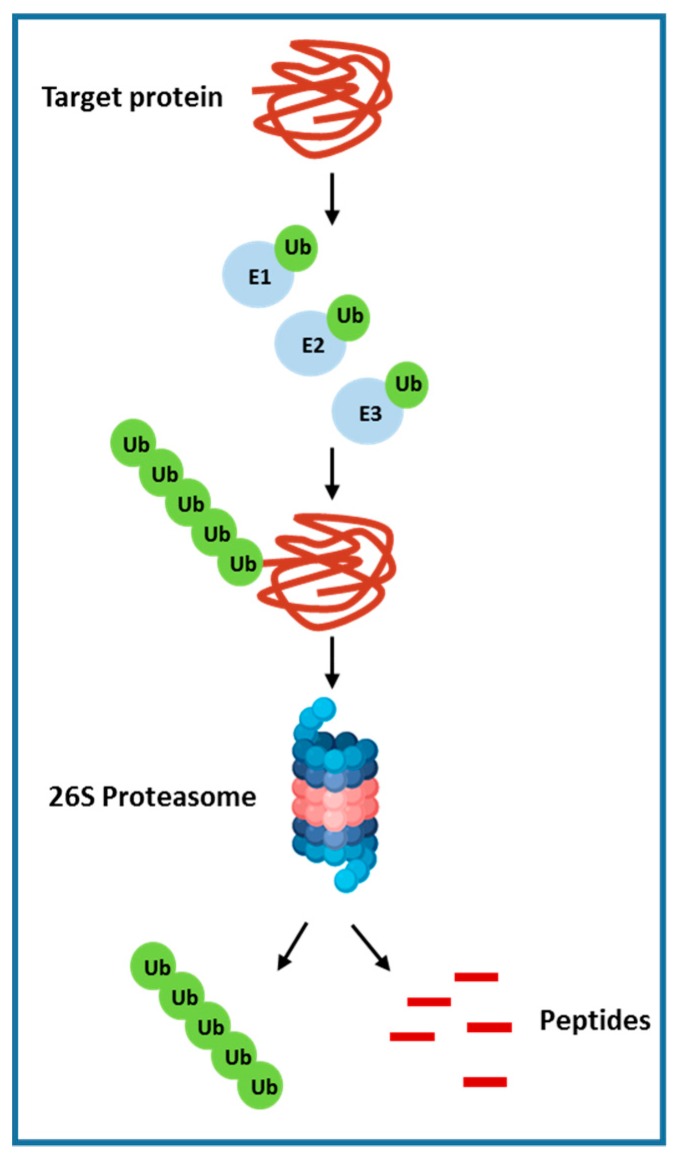
The Ubiquitin proteasome pathway.

The ubiquitin-proteasome system (UPS) is involved in cellular protein degradation and is highly conserved in eukaryotic systems [113]. This pathway plays a vital role in cell homeostasis that includes receptor signal transduction, cell cycle regulation, endocytosis and apoptosis. Around 80% ubiquitin labelled protein is degraded by the proteasome in the cytoplasm and nucleus. The rest of the 20%, which is mostly cell surface proteins, are absorbed by endocytosis and degraded in lysosomes. Ubiquitin-proteasome–mediated protein degradation is accomplished via sequential steps, which includes ubiquitin-activating enzyme (E1) and multiple ubiquitin-conjugating enzymes (E2s) and ubiquitin ligases (E3s). ATP-dependent transfer of ubiquitin to an E2 ubiquitin-conjugating enzyme is mediated by the ubiquitin-activating enzyme E1. Enzyme E2 then transfers the ubiquitin moiety to a downstream E3 ligase, which, in turn, ubiquitinates its specific substrate or substrates. This sequential addition of ubiquitin molecules to the nascent ubiquitin chain results in the formation of a polyubiquitin chain. These polyubiquitinated proteins are determined and degraded by the multisubunit 26S proteasome into peptide fragments and ubiquitin monomers, which are then recycled [19] (Figure 2). A variety of proteins related to cancer are regulated by the ubiquitin-proteasome system, which includes the cell surface tyrosine kinase growth factor receptors (EGFR), the tumor suppressors PTEN, p27, p53, and pRb; transforming growth factor b receptor (TGF-β) and other cell cycle regulators [114]. Recent studies have further elucidated the importance of E3 enzymes as compare to E1 and E2, which has only few reports focused on their roles. E3 ubiquitin ligases expression were found to be overexpressed in multiple human cancers such as lung and breast cancer. Furthermore, E3 ubiquitin ligase overexpression was allied with poor prognosis and reduced patient survival. Importantly, most of the E3 ligases play a key role in carcinogenesis and are required for the maintenance of the cancer cell phenotype. Additionally, in HCC, several cancer-related proteins interacts with enzymes of the ubiquitin-proteasome pathway and has shown to function as tumor suppressors. For example, the E3 ubiquitin ligase murine double minute 2 (MDM2) and BRCA1 both of which regulates p53 levels [115,116], another E3 ligase, Smad ubiquitination regulatory factor-2 (Smurf2), regulates Smad proteins and the TGF-b receptor complex levels [117]. Given the contribution of UPS in HCC, multiple compounds that targets the 26S proteasome complex or individual E3 ligase have been developed. Bortezomib or PS-341 (Velcade), a dipeptide boronic acid analogue, is the first FDA (Food and Drug Administration) approved compound that is a potent, selective and reversible inhibitor of 26 S proteasome. Bortezomib have shown to inhibit multiple proteins as it targets the final common effector in the ubiquitin proteasome pathway. Studies with this drug have shown to inhibit tumor growth and induce apoptosis of multiple cancer cell types and hence was approved for the treatment of late-stage multiple myeloma [19].

### 2.8. Hedgehog Signaling Pathway

Currently, the hedgehog (HH) signaling plays a crucial role in tissue pattern, cell differentiation and proliferation. In humans, the HH pathway is activated by three ligands: Sonic Hedgehog (SHH), Indian hedgehog (IHH) and Desert Hedgehog, of which SHH is the most studied [118,119]. HH pathway is composed of the Hedgehog ligand, nuclear transcription factors and two transmembrane protein receptors Patched-1 (Ptch1) and Smoothened (Smo) [56]. SHH ligands binds to the Ptch1 receptors and blocks the inhibition of Ptch-1 on Smo thereby activating the Hh signaling pathway. In the cytoplasm, Smo activates the downstream GLI transcription factors Gli1 and Gli2, known activators, as well as Gli3, a repressor [118], thereby regulate cell growth, differentiation and proliferation. In HCC, HH signaling pathway plays an important role in development and invasion and have been shown to be abnormally activated [120,121,122]. Additionally, the expression of transmembrane proteins, Ptch1 and Gli1, are detected in 50% of the human HCCs suggesting that this pathway is frequently activated in HCCs. The first study performed by Sicklick et al. in 2006 showed the activation of Hh signaling, as illustrated by the increased expression of Shh, Ptch, Smo and Gli1 in cancer cells, as compared to the respective non-tumors tissues. Additionally, increased levels of Smo was correlated with the increased size of HCC nodules [123]. In another similar study, HH activation was detected by analyzing the expression of Shh, Ptch, Smo and Gli1 in human HCC tumor tissues. Increased expression of these genes were detected in the tumor tissues, as compared to the adjacent normal tissue, further reinforcing the key role of Hh in HCC [124]. Furthermore, studies in liver cancer tissues demonstrated Gli2 gene ablation reduced the expression of c-myc and Bcl-2 and elevated the p27 expression which regulates the cell cycle, reduce proliferation and inhibits the growth of liver cancer cells [125]. Studies in Huh7, Hep3B and HepG2 HCC cells have shown Bufalin (Bu), one of the topoisomerase II inhibitors, influences the Gli1 and Gli3 expression of Hh signaling pathway and inhibits liver cancer cell proliferation, invasion and metastasis [58]. GDC-0449, antagonist of Smo, have shown to reduce the tumor size and significantly reduced cell filtration in HCC bearing mice [59] (Table 1). Considering its involvement in the initiation and development of HCC, Hh targeted therapies could be a promising strategy to combat HCC.

### 2.9. Liver Cancer Stem Cells

Targeting the tumor microenvironment is another issue. Even though the cells with self-renewing capacity in HCC is well supported, the origin of these cells is not clearly understood. Recently, growing evidence supports the novel notion that tumor initiation can be driven by a subset of cells with stem cell features. These cancer stem cells (CSCs) are responsible for not only tumor initiation, but also for tumor persistence, relapse, metastasis, chemoresistance and radioresistance [42]. This may explain why HCCs are usually resistant to conventional chemotherapies. Additionally, classical therapeutic regimens, as well as new generation therapies (e.g., Sorafenib), predominantly targets proliferating cells, which are unlikely to be CSCs, as evidenced by frequent tumor relapse after therapy [126]. This view of the need for identification and characterization of signaling pathways and biomarkers associated with CSC biology to develop novel molecular cancer therapeutics to treat HCC. Immunohistochemical analysis of 63 HCC tissue specimens revealed CD133, marker for CSCs, to be frequently present in HCC and its expression was found to be increased in 26 specimens (41.3%). Increased levels of CD133 in HCC was correlated with poor survival and high recurrence rate [127]. Another study identifies a novel mechanism in which IL-6 mediated activation of STAT3 signaling resulted in an upregulated expression of CD133 in HCC cells and promotes HCC progression [128]. Further understanding of the molecular pathways involved in the generation of tumorigenic CSCs and the role of CSCs in HCC pathogenesis will provide useful information for developing novel treatments to combat HCC.

### 2.10. Immune Suppression or Immune escape Mechanisms in HCC

Liver is a lymphoid organ that receives blood from both the systemic circulation and intestine via the hepatic artery and portal vein, respectively. Since the liver is constantly exposed to exogenous antigens it maintains a unique immunological tolerance against invading pathogens. In HCC, tumor microenvironment has a complex interplay of T cells, B-cells, macrophages and hepatic cells to evade immune surveillance by activating multiple cellular signaling cascades. In tumors, the presence of CD8^+^T cells is associated with better outcomes, whereas presence of regulatory T cells (Tregs) is correlated with poor prognosis [129]. In a study, Tregs have shown to be involved in the deterioration of anti-tumor effects of T-lymphocytes both in vitro and in vivo [130]. Another study in HCC patients have shown an increased number of circulating Tregs, suggesting a key role in HCC pathogenesis [130]. An alternative mechanism of immune suppression in HCC is via the increase in immunosuppressive cytokines, which includes interleukin-4 (IL-4), IL-5, IL-8, and IL-10, as well as suppression of immune activating cytokines: IL-1, TNF, and interferon gamma [131]. A study with 74 patients with unresectable HCC demonstrated that serum IL-10 concentration is contributing to poor prognosis and low anti-tumor immunity in patients [132]. Recently, modulation of tumor immunity by the programmed cell death-1 (PD-L1/PD-1) immune checkpoint pathway is identified as an alternate mechanism of immune evasion [133]. Immunohistochemical analysis in HCC patients showed increased expression of PD-1 and PD-L1 which promoted CD8+ T-cells apoptosis and contributes to tumor evasion [134]. Recently, blocking of checkpoint molecules that block immune response against tumor cells has being increasingly recognized as an effective therapeutic approach in cancer. In a study from phase I/II trial in advanced HCC patients, anti-PD-1 monoclonal antibody nivolumab has shown antitumor activity [135]. A deeper understanding of immunotherapy and immune tolerance of the liver will be instrumental for designing therapeutic targets to treat HCC.

## 3. Conclusions and Future Directions

HCC evidently has a complex pathogenesis with many critical signaling pathways being involved in its development. In addition to their independent signaling conduits, there are multiple levels of crosstalk interactions among these signaling pathways to regulate each other. With advances in our understanding of the molecular mechanism of tumorigenesis and in the development of the targeted therapeutics, we are at the beginning of a new era of applications of targeted therapeutics for the treatment of liver cancer. Different strategies that target these pathways showed a varied amount of success in the clinic. In addition, novel strategies have been investigated and raised the potential for more efficient therapies [136,137,138]. Since HCC is a complex disorder and leads to dysregulation of multiple molecular pathways, it would be great strategy to identify the regulatory proteins that could inhibit two or more major signaling pathways simultaneously. The use of combinatorial trials with two or three drugs may be needed that impact the complex network of signaling pathways to eventually control tumor growth and survival. However, the use of a combination therapy might raise issues such as tolerability and drug-drug interaction, and thus, alternate, targeting molecular agents that control multiple signaling pathways and have multiple modes of action would be better [139]. In addition, targeted drug delivery systems (DDS) have demonstrated a significant potential in HCC treatment by selectively delivering therapeutic drugs into tumor sites. DDS could be utilized to resolve the limitations of conventional chemotherapies. Even though there have been a lot of preclinical studies of DDS, targeted DDS for HCC has yet to be made for clinical use [140]. With further discovery and advances in research on all potential HCC targets, including immunological, molecular and translational, we can generate effective treatment strategies to combat HCC.

## Figures and Tables

**Table 1:** List of Molecular Targets and potential therapeutic agents used against HCC.

Targets	Therapeutic Agents	Phase	Reference
VEGFR, Ras/Raf/MEK/ERK, PDGFR, c-KIT, RET	Sorafenib	3	[40,41]
VEGFR	Ramucirumab	3	[42]
VEGFR, PDGFR, FGFR, RET, SCFR	Lenvatinib	3	[42]
VEGFR, PDGFR, BRAFFGFR, KIT, RET	Regorafenib	3	
EGFR/ErbB1/Her1	Erlotinib	3	[43]
PI3K/Akt/mTOR	Everolimus	3	
PI3K/Akt/mTOR	Sirolimus	3	[44]
c-MET	Cabozantinib	3	
VEGFR2, FGFR1	Brivanib	3	[45,46]
VEGFR, PDGFR, c-KIT, RET	Sunitinib	3	[47]
VEGFR, PDGFR	Linifanib	2	[48]
VEGFR, FGFR, PDGFR, c-KIT	E-7080	2	
VEGFR2, FGFR, PDGFR	TSU-68	2	
VEGFR2, MET, RET	XL-184	2	
VEGF	Bevacizumab	2	[49]
VEGFR, PDGFR, c-KIT	Cediranib	2	[50]
EGFR	Cetuximab	2	[42]
c-MET	Tivanitib	2	[51]
VEGFR, PDGFR, FGFR	BIBF-1120	2	[42]
VEGFR, EGFR	Vatalanib (PTK787)	2	[52]
IGF/IGFR	IMC-A12	2	[53]
IGF/IGFR	AVE1642	1	[54,55]
VEGFR, PDGFR, FGFR-1Raf, RET, c-KIT	BAY73-4506	1	[42]
IGF/IGFR	BIIB922		
MEK inhibitor	CI-1040		[19]
MEK inhibitor	PD 0325901		[19]
PI3K/Akt/mTOR	AZD8055		[56]
Wnt-β-catenin	PFK118-310		[56]
Wnt-β-catenin	PFK115-584		
Wnt-β-catenin	CGP049090		
26 S Proteasome	Bortezomib		[19]
STAT3	CIMO		[57]
Gli1 and Gli3	Bufalin		[58]
Smo	GDC-0449		[59]
